# Identification of necroptosis-related gene signatures for predicting the prognosis of ovarian cancer

**DOI:** 10.1038/s41598-024-61849-y

**Published:** 2024-05-15

**Authors:** Yuling Qin, Yawen Sheng, Mengxue Ren, Zitong Hou, Lu Xiao, Ruixue Chen

**Affiliations:** 1 Guang’anmen Hospital, China Academy of Chinese Medical Sciences, No. 5, Beixiange Road, Xicheng District, Beijing, 100053 China; 2 Shandong University of Traditional Chinese Medicine, Jinan, 250014 Shandong China

**Keywords:** Ovarian cancer, Necroptosis, Signatures, Prognosis, Biophysics, Cancer

## Abstract

Ovarian cancer (OC) is one of the most prevalent and fatal malignant tumors of the female reproductive system. Our research aimed to develop a prognostic model to assist inclinical treatment decision-making.Utilizing data from The Cancer Genome Atlas (TCGA) and copy number variation (CNV) data from the University of California Santa Cruz (UCSC) database, we conducted analyses of differentially expressed genes (DEGs), gene function, and tumor microenvironment (TME) scores in various clusters of OC samples.Next, we classified participants into low-risk and high-risk groups based on the median risk score, thereby dividing both the training group and the entire group accordingly. Overall survival (OS) was significantly reduced in the high-risk group, and two independent prognostic factors were identified: age and risk score. Additionally, three genes—C-X-C Motif Chemokine Ligand 10 (CXCL10), RELB, and Caspase-3 (CASP3)—emerged as potential candidates for an independent prognostic signature with acceptable prognostic value. In Gene Ontology (GO) and Kyoto Encyclopedia of Genes and Genomes (KEGG) enrichment analyses, pathways related to immune responses and inflammatory cell chemotaxis were identified. Cellular experiments further validated the reliability and precision of our findings. In conclusion, necroptosis-related genes play critical roles in tumor immunity, and our model introduces a novel strategy for predicting the prognosis of OC patients.

## Introduction

Ovarian cancer (OC) is frequently diagnosed at an advanced stage, which contributes to its high mortality rates. In 2020, there were approximately 314,000 new cases and over 207,000 deaths worldwide^1,2^. Currently, the most common treatments for OC are surgery and chemotherapy; however, the low survival and high recurrence rate continue to confound the medical community. Consequently, new treatment modalities, such as targeted therapy or immunotherapy, are imperative^3^. The purpose of this study was to develop a prognostic model for OC to facilitate more effective targeted therapies.

Necroptosis is a novel programmed cell death pathway characterized by significant inflammatory outcomes and immune responses. It differs from apoptosis, and its common symptoms include cellular swelling, organelle dysfunction, extensive mitochondrial damage, and plasma membrane rupture^4,5^. Necroptosis is dependent on receptor-interacting protein kinase 1 (RIPK1), RIPK3, and mixed lineage kinase domain like pseudokinase (MLKL) from a morphological standpoint^6,7^. According to studies, necroptosis may play crucial roles in tumorigenesis and also has tumor-suppressing effects^8,9^. However, the relationship between necroptosis and OC remains unclear. In order to predict the prognosis of OC, we performed a novel necroptosis-related gene signatures analysis. Using high-throughput sequencing technology and bioinformatics analysis, researchers can screen out a large number of Necroptosis-related Genes (NR Genes) from ovarian cancer tissues in recent research. These NR Genes are likely to be closely related to the occurrence and development of ovarian cancer. We can gain a deep understanding of their biological functions and prognosis in ovarian cancer through further functional annotation and enrichment analyses of these NR Genes. The advancement of interaction prediction research in various fields of computational biology would provide valuable insights into genetic markers related with OC in recent years^10,11^.

## Materials and methods

### Data collection

We acquired gene expression and clinical information of 379 cancer samples from TCGA (https://tcga-data.nci.nih.gov/tcga/). Raw TCGA data is subjected to an ID transformation. Then, genes associated with necroptosis were retrieved from previous research and the published literature. In addition, TCGA provides mutation data as supplementary information. CNV data were downloaded from the UCSC database (http://genome.ucsc.edu/). In addition, GSE140082 was downloaded from the Gene Expression Omnibus (GEO) database as an external cohort for validation.

### Visualization of necroptosis-related genes and CNVs

It's been demonstrated that CNVs can predict different tumor subtypes^12^. The collected CNVs genes exhibited copy loss and copy gain variants, and we then evaluated the CNVs at the level of the 23 chromosomes^13,14^. Mutation annotation format (MAF) data were processed and analyzed using the MutSigCV algorithm and the"mafools" software package. A waterfall plot was used to visualize the mutational information of NR Genes from OC patients in the TCGA database. The CNV frequency figures were visualized using the "barplot" command in the R language. The ordinate represented the frequency of the CNV that corresponded to NR Genes, and the abscissa represented the name of NR Genes. Circles of CNV frequencies were drawn using the "RCircos" package (Red: high frequency of increased NR Genes copy number; Blue: high frequency of deleted NR Genes copy number).

### Consensus clustering analysis

Consensus clustering analysis of OC samples was performed using both "limma" and "consensusClusterPlus" packages based on euclidean distance and Wards linkage. Initially, 379 cancer samples were divided into k-clusters. The quantitative stability evidence was obtained based on cumulative distribution function (CDF) in order to further confirm the optimal number of clusters. Next, the identified k clusters and actual patient prognosis were evaluated using the Kaplan–Meier and progression-free survival curves. Based on the sample expression level of OC patients in the TCGA database, the matrix scores and immunity scores were calculated using the “ESTIMATE” package among genotypes. In order to quantify and analyze the immune cell infiltration levels of each genotyping sample, the “CIBERSORT” computational R package was used to plot heat maps and pairwise difference plots. Gene function and differentially expressed genes studies were performed for every cluster. The ClueGo plugin in Cytoscape (3.9.0) was used to do a functional analysis of DEGs, using a threshold of p < 0.05.

### Establishment and validation of the risk signature

Half of the 379 cancer samples were chosen at random as the train group using Strawberry Perl and the caret R package. Initially, a Univariate Cox analysis was performed on the necroptosis-related gene signature in the train group to identify prognosis-related genes (p < 0.05)^15,16^. Then, the optimal Log(λ) value was determined using LASSO regression analysis with tenfold cross-validation^17–22^, and a gene signature was constructed accordingly^23,24^. To validate our signature, the entire group was utilized in order to confirm the prognostic signature. Risk score $\sum_{i=1}^{n}expi\times ci$=$\sum_{i=1}^{n}expi\times ci$ (where n, expi, and ci represent the number of prognostic genes, the expression value, and the coefficient of gene I respectively. The patients were then split into high-risk and low-risk groups based on the median value of the risk score, the OS of the two risk subgroups was analyzed and compared using the "survival" package. In addition, Kaplan–Meier curves and multivariable analyses identified independent prognostic factors^25,26^. A risk heat map, a risk curve, ROC curve analysis^27,28^, and survival analysis were performed for our signature^29,30^. For extra validation, we also used an independent external dataset (GSE140082). GO/KEGG enrichment analysis^31–33^ of DEGs was performed using the clusterProfiler R package^34,35^, and we used gene set enrichment analysis (GSEA) to investigate significantly enriched pathways in the high-risk and low-risk groups. Additionally, we contrasted m6A-related genes, immune activity, survival rates, and other factors.

### Cell culture

Sk-ov-3 human ovarian cancer cell line, purchased from Shanghai Fu Heng Biological Co. The cells were cultured in DMEM + 10% FBS medium (both purchased from gibco) at 37℃, 5% CO2, pH 7.2–7.4, at a sterile constant temperature. The cells were passaged when the cell fusion reached 90%.

### CCK8

Necrostatin-1 (Nec-1) is a potent inhibitor of necroptosis, purchased from MedChemExpress. The sk-ov-3 cells were inoculated 24 h in advance in 96-well plates at a density of 5000 cells/well, and after one day and night of complete cell wall attachment, the cells were replaced with serum-free medium with or without 100 nM Nec-1 and incubated for 24 h with the cck8 Cell Activity Kit ( The absorbance at 450 nm was measured using the cck8 cell activity kit (purchased from Shanghai Toyobo Biotechnology Co., Ltd.).

## Results

**Figure 1:**
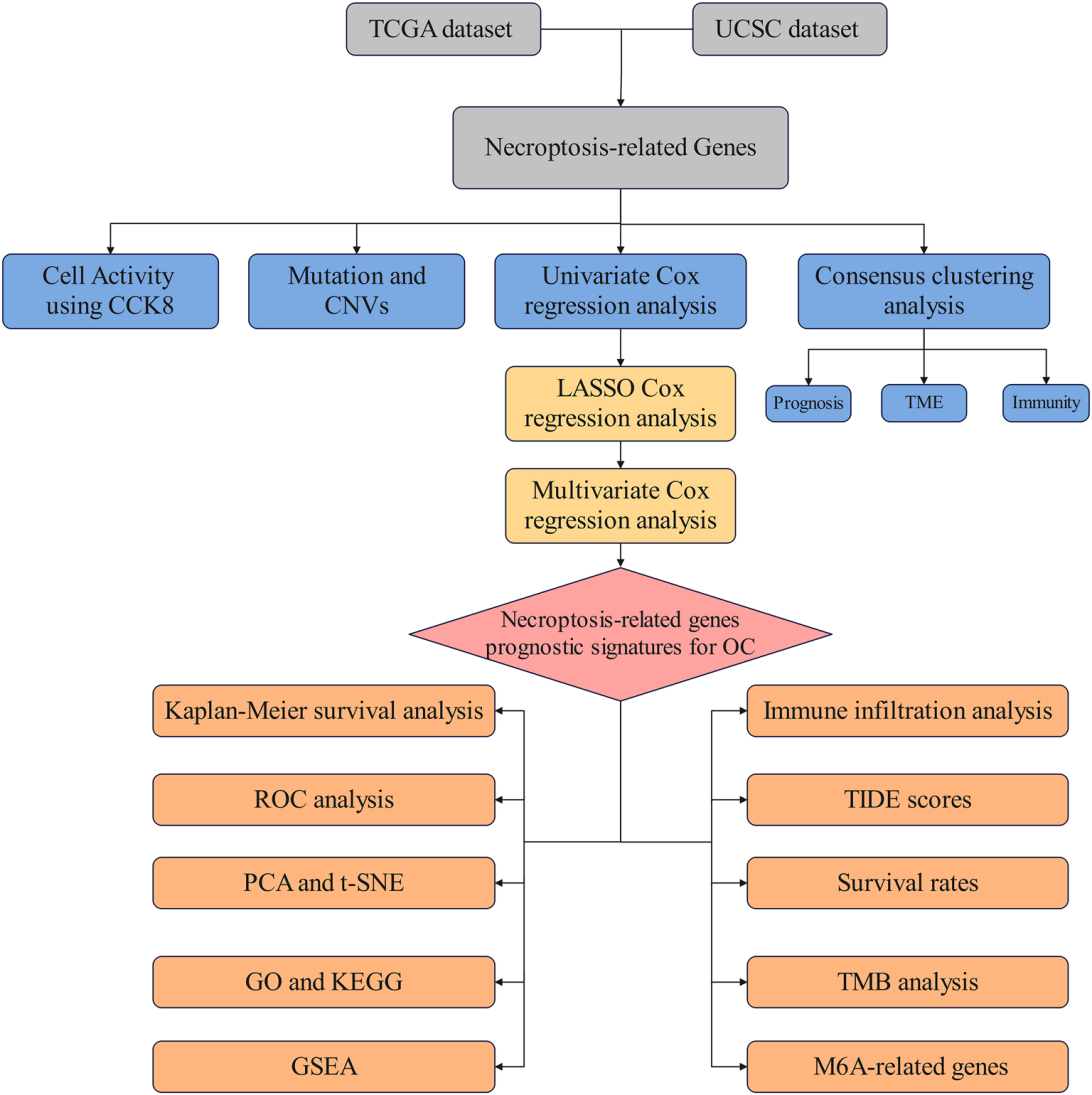
Flow diagram of full-text data.

The research fow of this paper is shown in Fig. 1.

### Necroptosis-related genes and CNVs in OC patients

**Figure 2:**
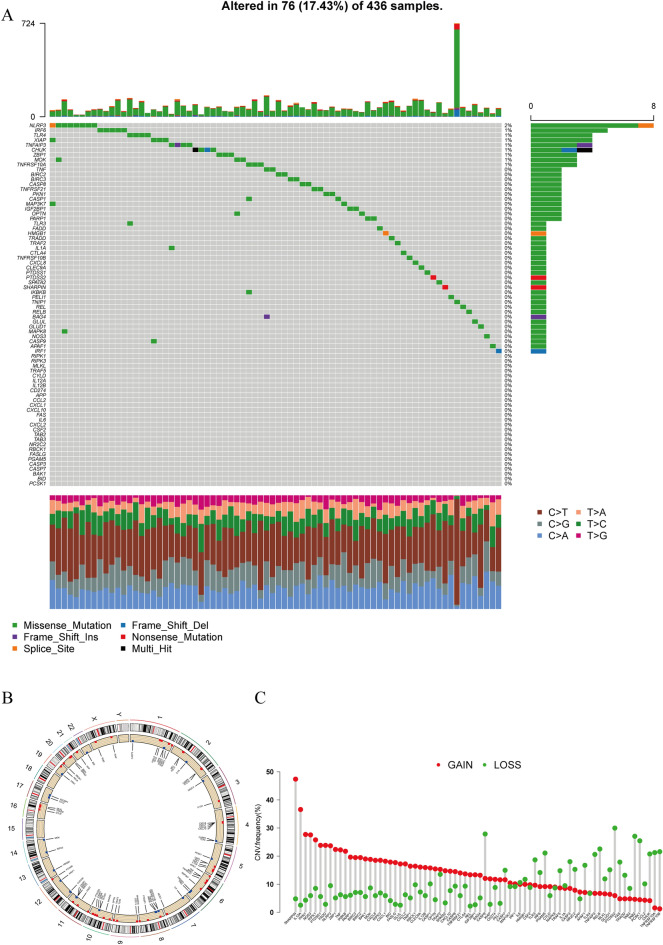
Genes and CNVs associated with necroptosis in OC. (**A**) Identified 76 genes related to necroptosis in OC samples. (**B**) The position of variations on 23 chromosomes. (**C**) The frequency of CNV (gain/loss) status.

Finally, 379 cancer samples from TCGA and UCSC were used to extract 76 necroptosis-related genes, including NLRP3, TLR4, and IRF6 (Fig. 2A). Then, we calculated the frequency of CNV status (gain/loss) and discovered that CNV loss was more prevalent overall (Fig. 2C) (red: gain; green: loss). CNV is a structural variation in chromosome that can lead to amplification or deletion of a section of chromosome; therefore, to pinpoint the location of variations on 23 chromosomes, a heatmap was created (Fig. 2B). In the graph, CNVs are displayed at a maximum of the region of human chromosomes 1 and 6.

### Cancer classification

**Figure 3:**
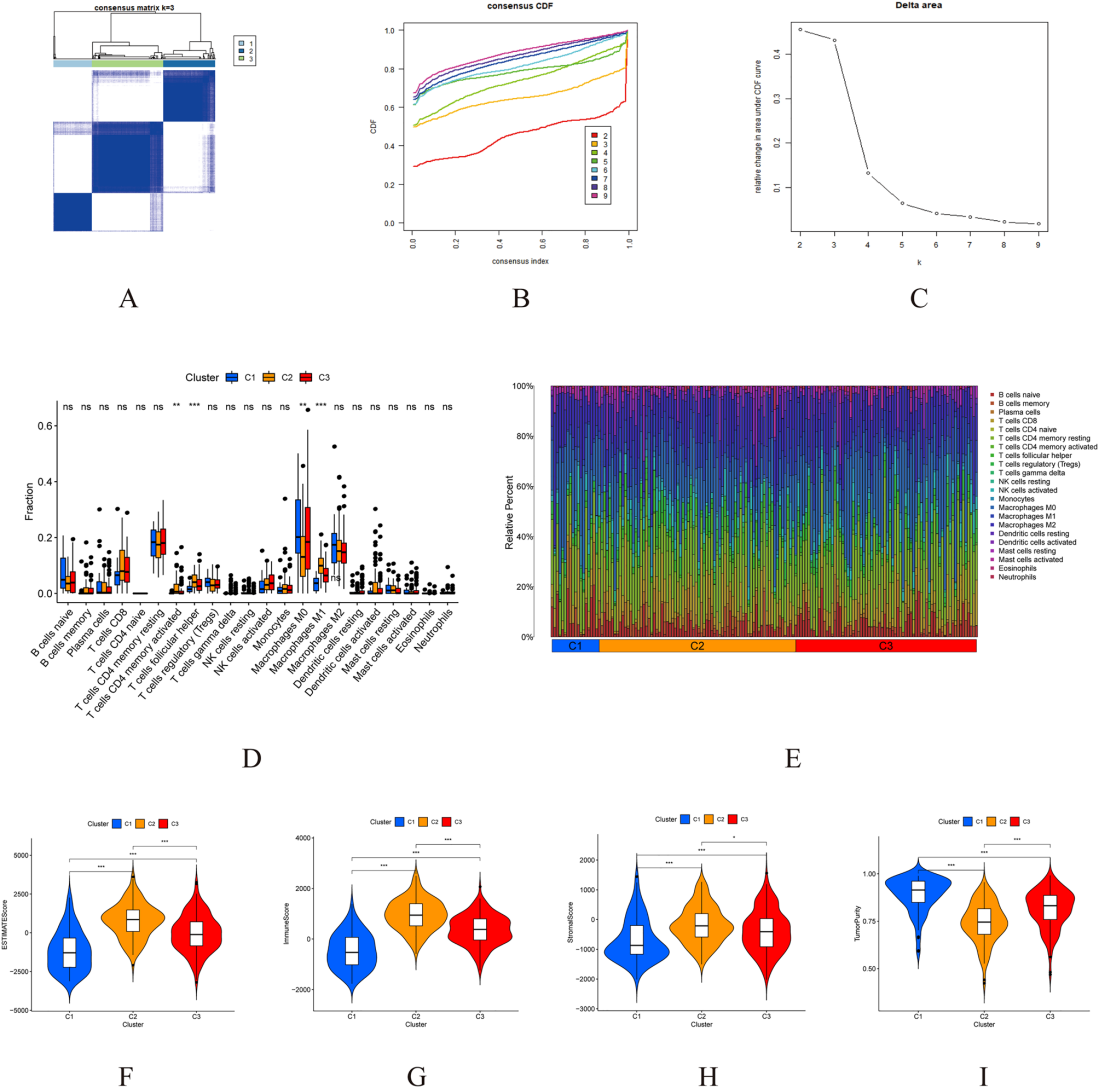
Comparisons of the 76 necroptosis-related genes into three clusters. (**A**–**C**) Using the CDF to determine K-cluster. (**D**) Immune cell expression in three clusters. (**E**) The proportion of various immune cells present in these clusters. (**F**–**I**) TME scores of three clusters.

**Figure 4:**
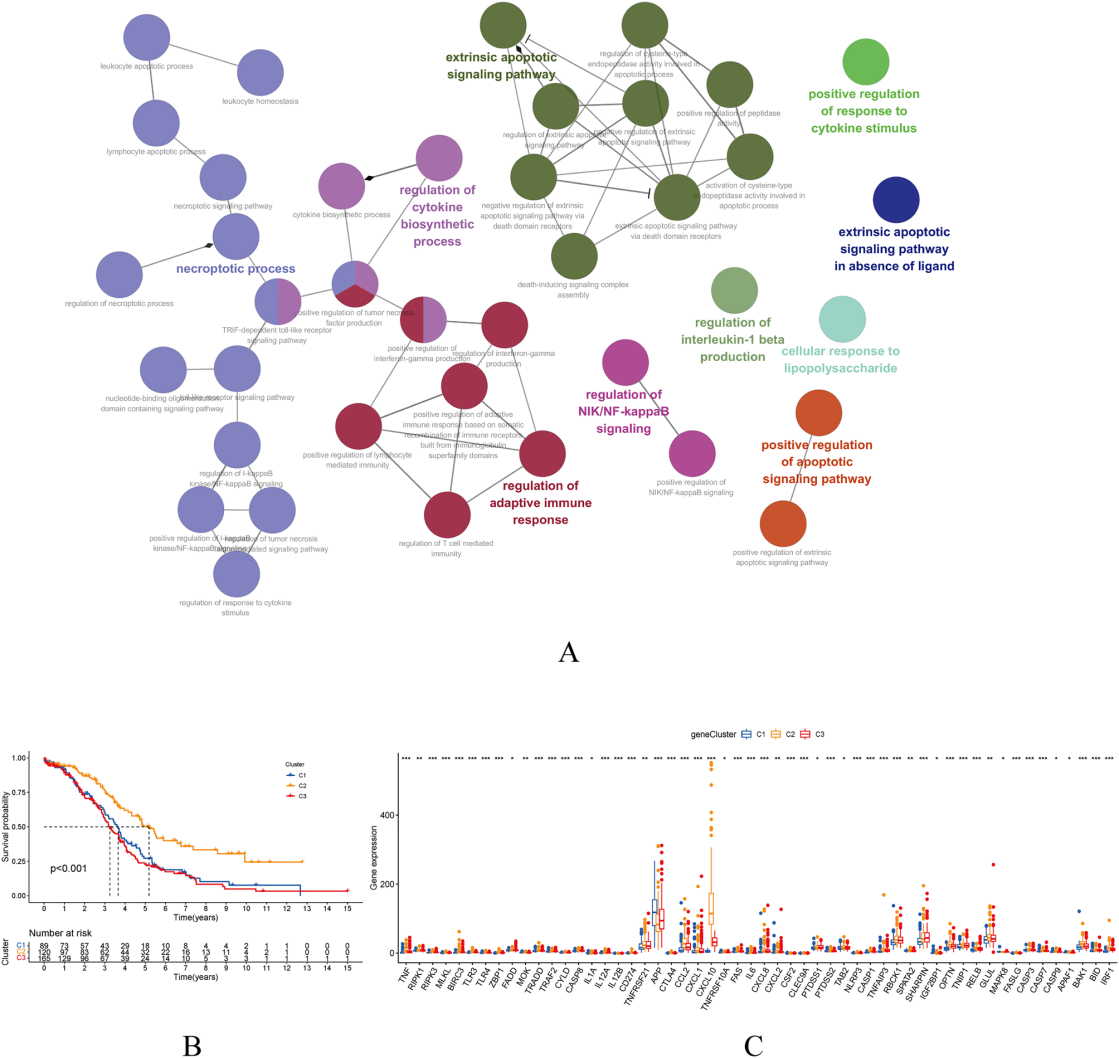
(**A**) Functional Analysis of the differentially expressed genes (DEGs) of three clusters. (**B**) Probability of survival in the three clusters. (**C**) The levels of gene expression in three clusters.

Using the CDF of the consensus index, we calculated the consensus matrix for each value of k by increasing the clustering variable (k) from 2 to 9. This allowed us to use all 379 OC patients from the TCGA to define the associations between the expression of 76 genes related to necroptosis and OC classifications. The greatest value, k = 3, showed that 379 OC patients could be grouped into three groups (Fig. 3A–C). Figure 3D illustrates the minimal variations in immune cell expression levels among the three clusters, while Fig. 3E displays the percentage of unique immune cells inside each cluster. Tumor purity and TME scores (immune, stromal, and estimation scores) for three clusters are shown in Fig. 3F–I. Figure 4A displays the functional analysis results of a differentially expressed genes conducted to better understand the differences among three clusters. According to the Kaplan–Meier analysis (Fig. 4B, p < 0.001), cluster 3 had a significantly better survival probability than the other clusters. In cluster 3, CXCL10 expression was particularly high, while no other gene showed any specificity (Fig. 4C).

### Construction and verification of the model

**Figure 5:**
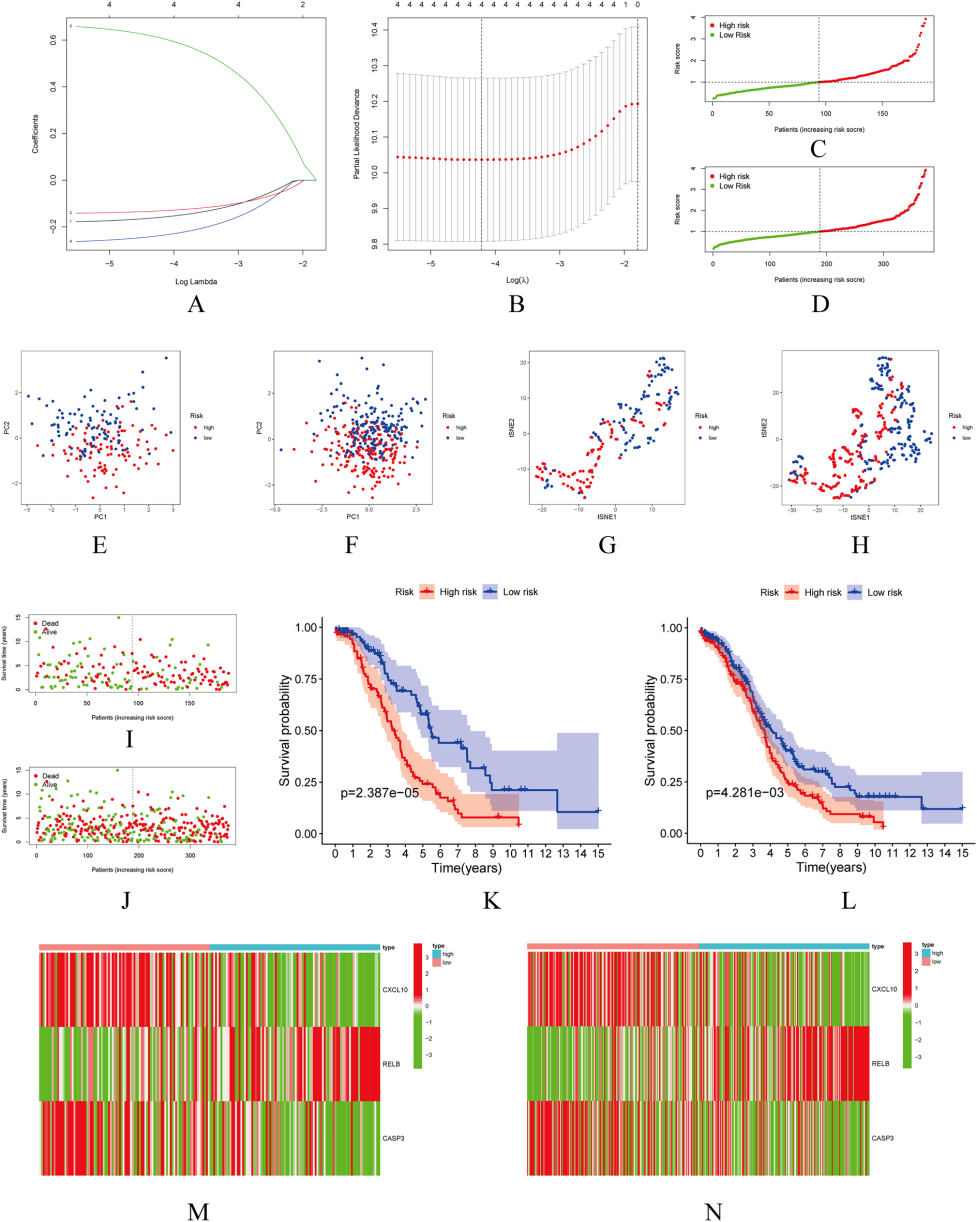
Demonstrates the construction and prognostic value of the three necroptosis-related gene models in the train and entire groups. (**A**) Cross-validation performed 10 times for variable selection in the LASSO model. (**B**) LASSO coefficient profile of three genes associated with necroptosis. (**C**,**D**) Risk scores for the train and the entire group. (**E**,**F**) The train and entire groups' principal component analysis (PCA). (**G**,**H**) The tSNE analysis, between the train's high- and low-risk groups and the entire group. (**I**,**J**) Survival rate of OC patients in the train and the entire group. (**K**,**L**) OS (survival probability) Kaplan–Meier survival curves between high- and low-risk groups in the train and the entire group. (**M**,**N**) Expression of three relevant genes in the train and the entire group.

On the basis of clinical OC cases from the TCGA, prognosis-related genes were analyzed using univariate Cox proportional hazard regression analysis (p < 0.05). Due to the possibility of overfitting problems in the prognostic model, the Lasso regression with tenfold cross-validation was employed (Fig. 5A,B). The following formula was used to calculate the risk score for OC patients: risk score = CXCL10 × (− 0.186741112236078) + RELB × (0.669433208748248) + CASP3 × (− 0.285126720445466).

A comparison of risk scores, principal component analysis (PCA), tSNE analysis, survival time, and Kaplan–Meier survival curves of OS (survival probability) of OC patients were made between high- and low-risk groups in the train and the entire group (Fig. 5C–L). The median risk score was utilized to create high- and low-risk subgroups. The group with the lowest risk had a better prognosis than the groups with the highest risk, according to these figures.. Relevant gene expression differences between the two groups were also observed (Fig. 5M,N). Furthermore, We added an additional validation set (GSE140082) with a survival curve illustrating that the high-risk group is detrimental to patient prognosis (Supplementary Fig. 1) and a ROC curve illustrating its good predictive value (Supplementary Fig. 2).

### Construction of nomogram

**Figure 6:**
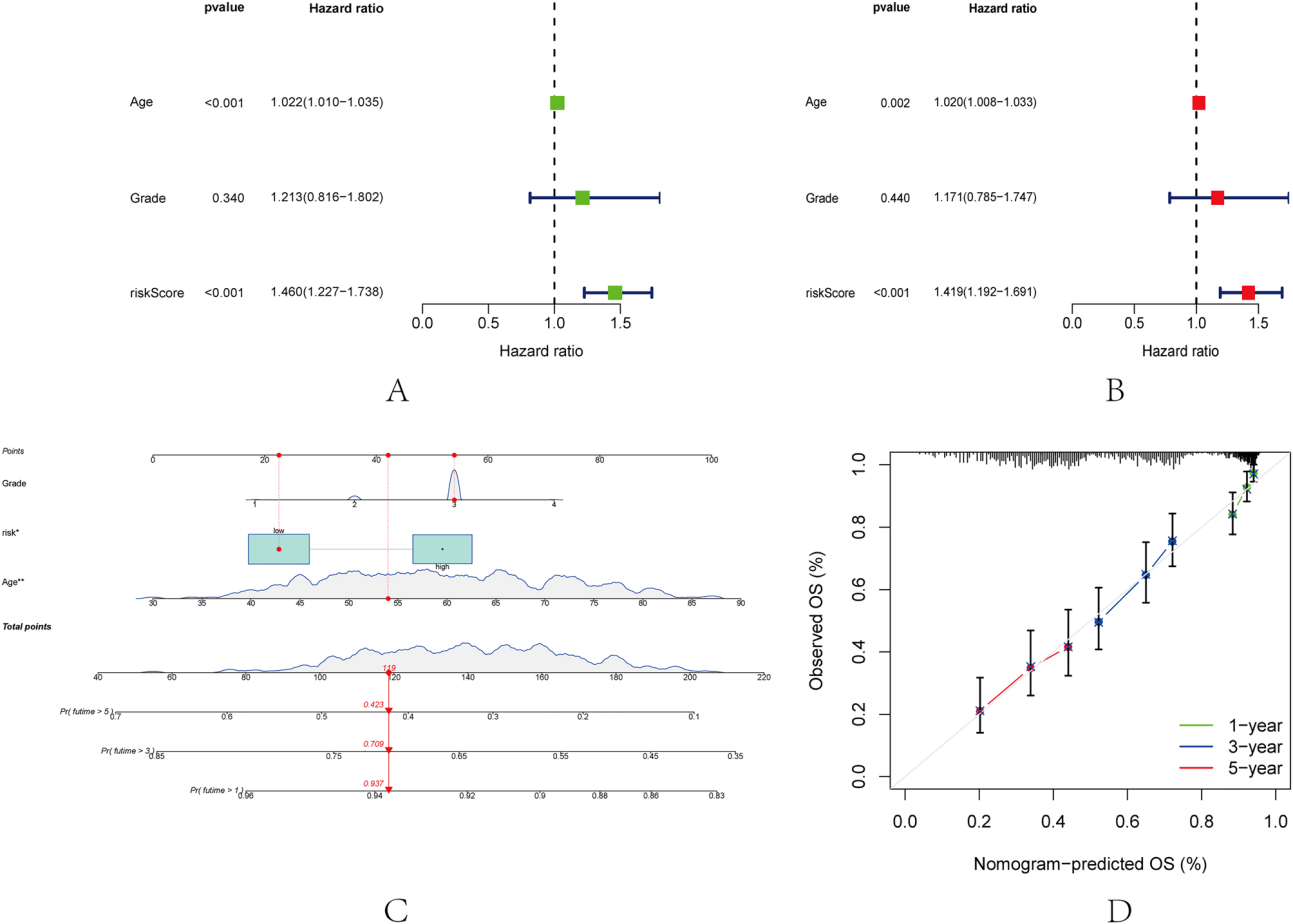
The risk model nomogram. (**A**,**B**) Univariate and multivariate Cox regression analyses of clinical factors (age, grade) and risk score. (**C**) Nomogram incorporating independent variables (risk score and age) for predicting the 1-, 3-, and 5-year incidences of OS in OC patients. (**D**) 1-year, 3-year, and 5-year OS calibration plots.

It was discovered that age and risk score were independent prognostic factors. The hazard ratio (HR) and 95 percent confidence interval (CI) for age and risk score are as follows:: risk score 1.460 (1.227–1.738) (p < 0.001) in univariate Cox (uniCox) regression and 1.419 (1.192–1.691) (*p* < 0.001) in multivariate Cox (multi-Cox) regression, respectively; age in uniCox regression (1.022 and 1.010–1.035; *p* < 0.001) and age in multivariate Cox regression (1.020 and 1.008–1.033; *p* = 0.002) (Fig. 6A,B). Tumor grade was excluded as a prognostic factor since its p value was not less than 0.05.

Two independent factors (risk score and age) were used to develop a nomogram for predicting the 1-, 3-, and 5-year OS incidences of OC patients (Fig. 6C). We also constructed 1-, 3-, and 5-year calibration plots to confirm that the nomogram accurately predicted 1-, 3-, and 5-year OS (Fig. 6D).

### Independent prognostic value of the risk model

**Figure 7:**
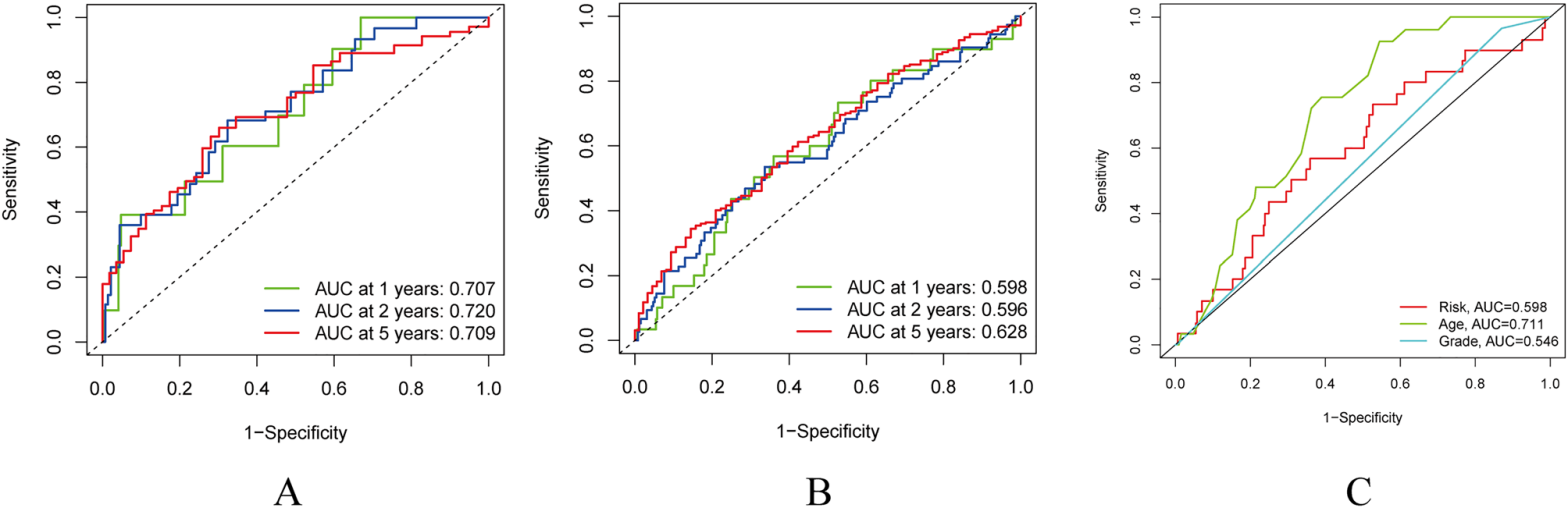
Evaluation of the risk model. (**A**,**B**) The area under the ROC curve (AUC) at 1 year, 2 years, and 5 years of the train and entire groups. (**C**) The risk score, age, and grade ROC curves.

The univariate Cox regression analysis and multivariate Cox regression analysis were performed to evaluate whether the prognostic risk score model could be used as an independent prognostic predictor. Both univariate Cox regression analysis and multivariate Cox regression analysis showed that the age, grades, and risk scores can be used as independent prognostic factors when assessing patients with OC. ROC curves were generated to validate the accuracy of the prognostic model, and the area under the ROC curve (AUC) could be used to illustrate the results of ROC curves^36^. The 1-, 2-, and 5-year AUC for the train group were 0.707, 0.720, and 0.709, respectively, while those for the entire group were 0.598, 0.596, and 0.628, respectively (Fig. 7A,B). Clinical ROC curve analysis proved that the clinical factor: age (0.711) has greater predictive ability than other clinical factors (Fig. 7C).

### Functional analyses of DEGs and gene set enrichment analysis (GSEA)

**Figure 8:**
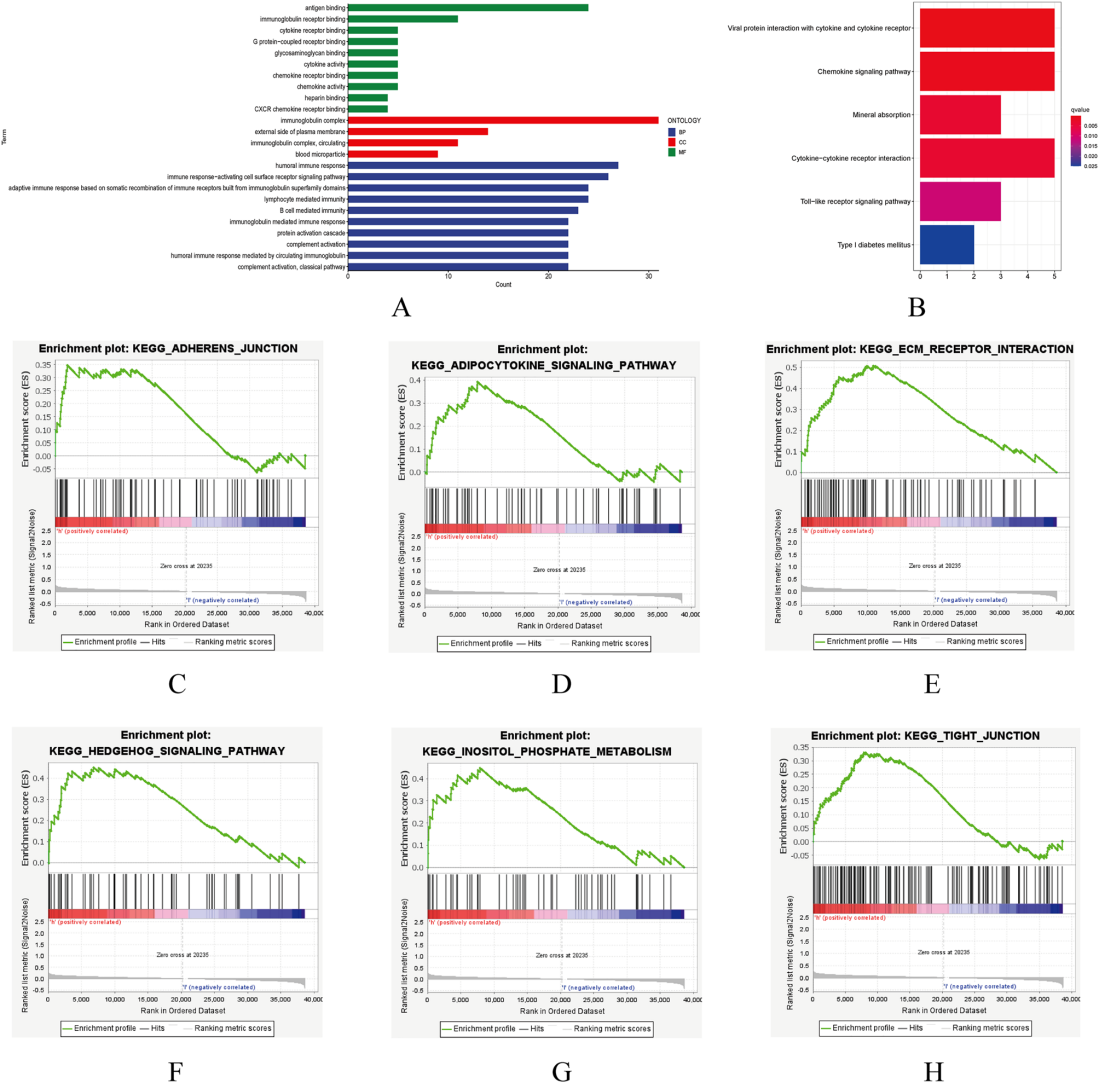
Functional analyses of DEGs and enrichment analysis of gene sets (GSEA). (**A**,**B**) Analysis of KEGG and GO enrichment pathways for DEGs in high- and low-risk groups. (**C**–**H**) The GSEA comparisons between high-risk and low-risk groups in the KEGG pathway for the entire group.

A differential gene KEGG and GO pathway enrichment analysis was conducted to gain a more comprehensive comprehension of the differences in gene functions and pathways between the high-risk and low-risk groups of the entire group. The DEGs were primarily associated with "immunoglobulin complex," "antigen binding," "immune response," "viral protein interaction with cytokine and cytokine receptor," "chemokine signaling pathways," and "cytokine-cytokine receptor interaction," according to our findings (Fig. 8A,B).

Additionally, we used GSEA to compare the two groups within the KEGG pathway for the entire group. In consequence, "adherensjunction", "adipocytokine", "ecm receptor interaction" "hedgehog signaling pathway", "inositol phosphate metabolism", and "tight junction" were significantly activated in high-risk patients (Fig. 8C–H).

### Immune activity in risk groups and m6a-related genes

**Figure 9:**
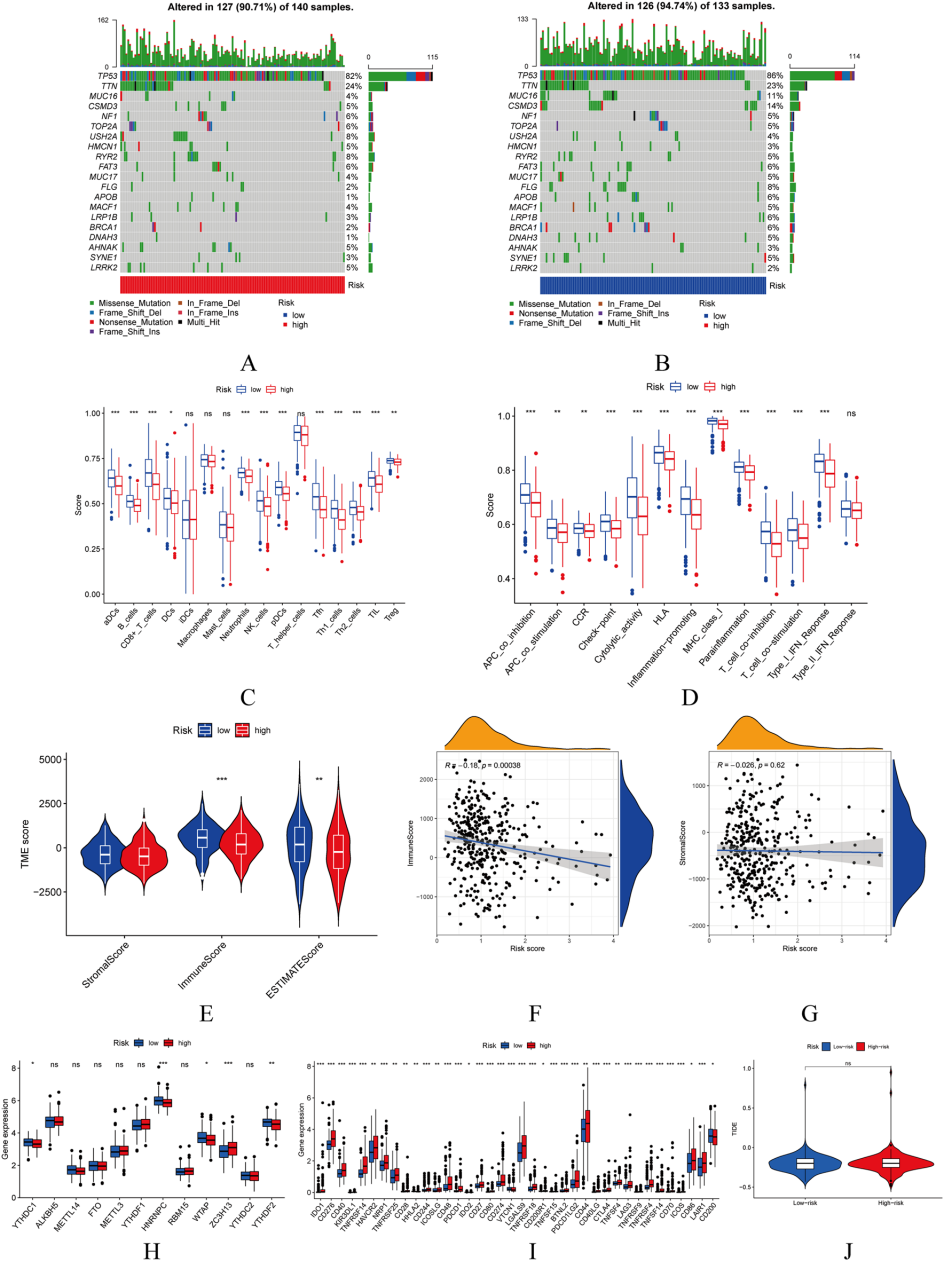
Immune activity in risk groups. (**A**,**B**) Variations in mutations between high- and low-risk groups. (**C**,**D**) Immune cell infiltration varies between high- and low-risk groups. (**E**) TME scores in the high- and low-risk groups. (**F**,**G**) Immune score and stromal score expression levels in high- and low-risk groups. (**H**–**J**) M6A-related genes, immune checkpoint expression, and TIDE scores in the high- and low-risk groups.

In the high-risk and low-risk groups, respectively, the two genes with the highest number of mutations were TP53 and TTN (Fig. 9A,B).

Immune cell infiltration plays a crucial role in tumor progression^37–39^. Using the single-sample gene set enrichment analysis(ssGSEA), we compared the level of immune-related pathways and functions between the high- and low-risk TCGA groups. Except for the induced dendritic cells (iDCs), the results showed that the high-risk group had nearly lower levels of immune cell infiltration than the low-risk group (Fig. 9C). In addition, there were notable differences between the two groups in terms of APC coinhibition, APC costimulation, cytokine-cytokine receptor (CCR), checkpoint, cytolytic activity, human leukocyte antigen (HLA), inflammation-promoting, MHC class I, parainflammation, T-cell coinhibition, T-cell costimulation, typeIFN response and typeIIFN response (Fig. 9D).

In addition, TME (including immune, stromal, and estimate scores) and m6A-related genes play a crucial role in the progression of tumors^40,41^. The TME scores in the high-risk group were lower, as shown in Fig. 8E. And we found that expression levels of immune scores had a negative correlation with risk score (p < 0.05), whereas stromal scores had little bearing on risk score according to our risk model (Fig. 9F,G).

Regarding m6A-related genes, the high-risk group had higher levels of METTL3, YTHDF1, RBM15, and ZC3H13 than the low-risk group (Fig. 9H).

Except for CD200, the expression of all immune checkpoints was nearly greater in the high-risk group than in the low-risk group (Fig. 9I). In addition, the TIDE score could simulate tumor immune evasion, which was more prevalent in the high-risk group compared to the low-risk group (Fig. 9J).

### CCk-8 results

**Figure 10:**
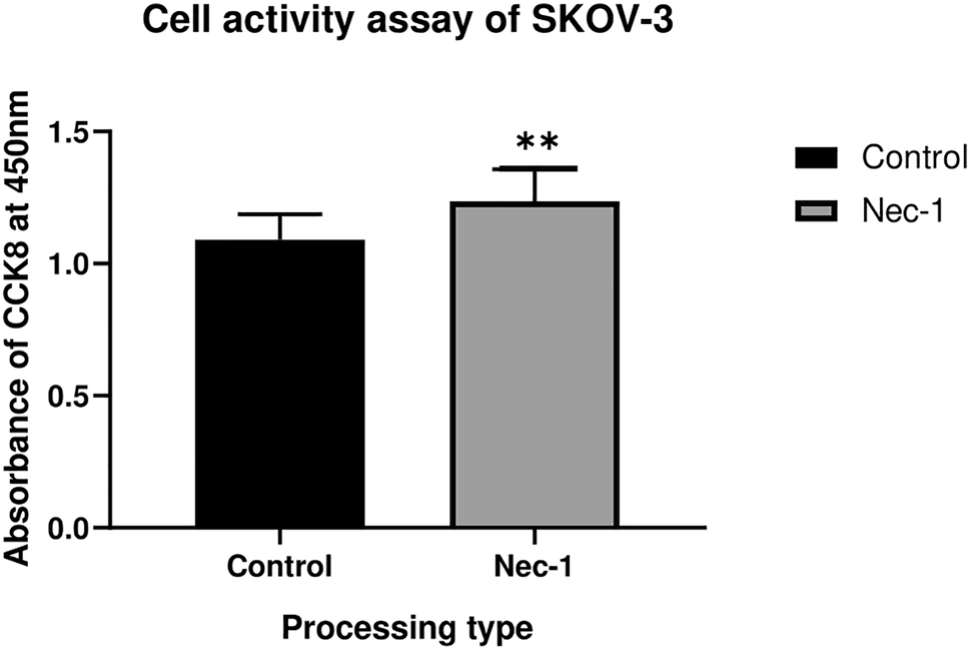
The Cell activity of SKOV-3 cells using the absorbance of CCK8 at 450 nm after the addition of Nec-1.

In addition, we used cck8 Cell Activity Kit to evaluate the activity of ovarian cancer cells by the appended Nec-1. CCk-8 results showed an increase in absorbance at 450 nm and enhanced activity of ovarian cancer cells after the addition of Nec-1, an inhibitor of necrotic apoptosis (Fig. 10).It seems reasonable to speculate that necroptosis may play crucial roles in the progress of OC.

## Discussion

OC is a malignant gynecologic tumor with a poor prognosis and a high mortality rate, and surgery is generally the first-line treatment^42^. Patients with extensive metastatic deposits and malignant ascites in the enterocoelia, which cannot be treated surgically, are frequently not diagnosed until the disease has progressed to an advanced stage^43,44^. The 5-year survival rate for patients with advanced-stage ovarian cancer is less than 50%^45^.

Necroptosis is a type of programmed cell death that is caspase-independent and distinct from apoptosis. It is mediated by death receptors such as receptor-interacting protein kinase (RIP) 1, TNF receptor 1, and RIP3, which cause necroptosis by activating the kinase domain-like protein (MLKL) to form necrosomes^46–48^.

Bioinformatics technology has been steadily advancing and developing over the past few years. Prognostic prediction of cancer by building the prognostic model contributes to the clinical treatment of patients. Studies have demonstrated that necroptosis can inhibit the development and occurrence of tumors, particularly colorectal cancer and breast cancer^49^. Additionally, it can promote inflammatory death and create a microenvironment conducive to the growth of tumor cells, thereby promoting tumor growth. According to research, RIPK1 inhibitors can inhibit tumor growth and limit metastasis^50,51^. Overall, necroptosis has been shown to be a double-edged sword for tumor progression and a potential tumor therapy, specifically for drug-resistant tumors^52^. Our goal is to create a new prognostic model to evaluate the prognostic significance of genes related to necroptosis in OC.

Before their differences were identified by Kaplan–Meier analysis and differential gene expression analysis, 379 cancer samples in this study were divided into three clusters. Afterward, we constructed a prognostic model based on 76 necroptosis-related genes isolated from OC patients. Half of the patients were randomly assigned to the train group, and the entire group was used to validate. The comparisons revealed that the low-risk group had a more favorable prognosis than the high-risk group. After calculating each patient's risk score, two independent prognostic factors (risk score and age) were identified, and univariate Cox (uni-Cox), multivariate Cox (multi-Cox), nomogram, and calibration curves were used to validate and evaluate the model.

Three necroptosis-related genes, CXCL10, RELB, and CASP3, were identified in the prognostic model through multiple regression analyses. CXCL10 is one of the inflammatory chemokines, and the research has shown that CXCL10 expression is crucial for ovarian cancer prognosis and TME immune characteristics^53^. Furthermore, CXCL10 was found to have an impact on angiogenesis in addition to stimulating T lymphocytes' ability to fight malignancies in the body^54^. According to some studies, CXCL10 regulates immunotherapy-sensitive tumors and is regarded as a biomarker of favorable prognosis^55^. In some studies, the level of RElB expression was associated with glioma, non-small cell lung cancer (NSCLC), and prostate cancer^56–58^. It has been demonstrated that CASPs, a group of proteases with similar structures, are involved in cell growth, cell differentiation, and apoptosis. Results indicated that CASP3 expression may improve the OS of patients with gastric cancer (GC)^59^. CASP3 is rarely expressed in various types of cancer, and CASP3 deficiency can cause cells to become resistant to microenvironmental stress and therapy^60^. However, additional research is necessary to determine how these genes interact during necroptosis.

The three necroptosis-related genes were used to construct a prognostic model with a decent prognostic value (0.707%, 0.720%, and 0.709% at 1, 2, and 5 years, respectively), and they can be considered prognostic biomarkers for OC. The GO and KEGG enrichment analyses revealed that the pathways were primarily associated with immune responses and inflammatory cell chemotaxis; therefore, it is reasonable to assume that necroptosis can regulate the infiltration of immune cells and the composition of the tumor immune microenvironment^61^.

### Limitations and future directions

Our study had limitations that must be taken into account. Firstly, the number of patients used in the study is small and there is a lack of health samples. The accuracy of our model needs to be validated in other external datasets and large clinical cohorts.

Computational biology has been evolving rapidly in recent years^62,63^, the advancement of interaction prediction research in various fields of computational biology would provide valuable insights into genetic markers and related diseases^64,65^, such as miRNA-lncRNA interaction prediction^66,67^. MicroRNAs (miRNAs) or long non-coding RNAs (lncRNAs) play important roles in biological activities, recent research has found that predicting the interaction between miRNA and lncRNA is the primary task for elucidating functional mechanisms^68,69^. In addition, ODE-based theoretical modeling studies on gene/protein signaling networks have been equally important for the study of understanding regulatory mechanisms and finding potential therapeutic targets in diseases^70–72^.

## Conclusion

In conclusion, the purpose of our study was to develop a prognostic model that would facilitate the formulation of clinical treatment decisions. We constructed a novel prognostic signature using three genes related to necroptosis in order to predict the prognosis of OC patients using multi-angle verifications, but the model did not have a very good predictive value. In the future, there is much work and further studies to be done in order to predict the prognosis and develop an accurate therapeutic strategy for the disease.

### Supplementary Information

Supplementary Legends.

Supplementary Figure 1.

Supplementary Figure 2.

## Data Availability

Our data comes from public databases The Cancer Genome Atlas(TCGA) (https://portal.gdc.cancer.gov/). All the data in this paper support the results of this study.

## Abbreviations

OC: Ovarian cancer
TCGA: The Cancer Genome Atlas
CNV: Copy number variation
UCSC: University of California Santa Cruz
DEGs: Differentially expressed genes
TME: Tumor microenvironment
OS: Overall survival
CXCL10: C-X-C Motif Chemokine Ligand 10
CASP3: Caspase-3
GO: Gene ontology
KEGG: Kyoto encyclopedia of genes and genomes
RIPK: Receptor-interacting protein kinase
MLKL: Mixed lineage kinase domain like pseudokinase
NR Genes: Necroptosis-related genes
GEO: Gene expression omnibus
CDF: Cumulative distribution function
GSEA: Gene set enrichment analysis
