# Supplementary material for: Identification of necroptosis-related gene signatures for predicting the prognosis of ovarian cancer
Source: Sci Rep. 2024 May 15;14:11133. doi: 10.1038/s41598-024-61849-y (PMC11096311; doi:10.1038/s41598-024-61849-y)
Supplement: Supplementary file 1 — Supplementary Legends. [file 41598_2024_61849_MOESM1_ESM.docx]

**Supplementary Figure 1：**Survival curve of the additional validation set (GSE140082).

**Supplementary Figure 2：**The 1-, 3-, and 5-year ROC curves of the additional validation set (GSE140082).
