# Supplementary figures and images for: Identification of necroptosis-related gene signatures for predicting the prognosis of ovarian cancer
Source: Sci Rep. 2024 May 15;14:11133. doi: 10.1038/s41598-024-61849-y (PMC11096311; doi:10.1038/s41598-024-61849-y)

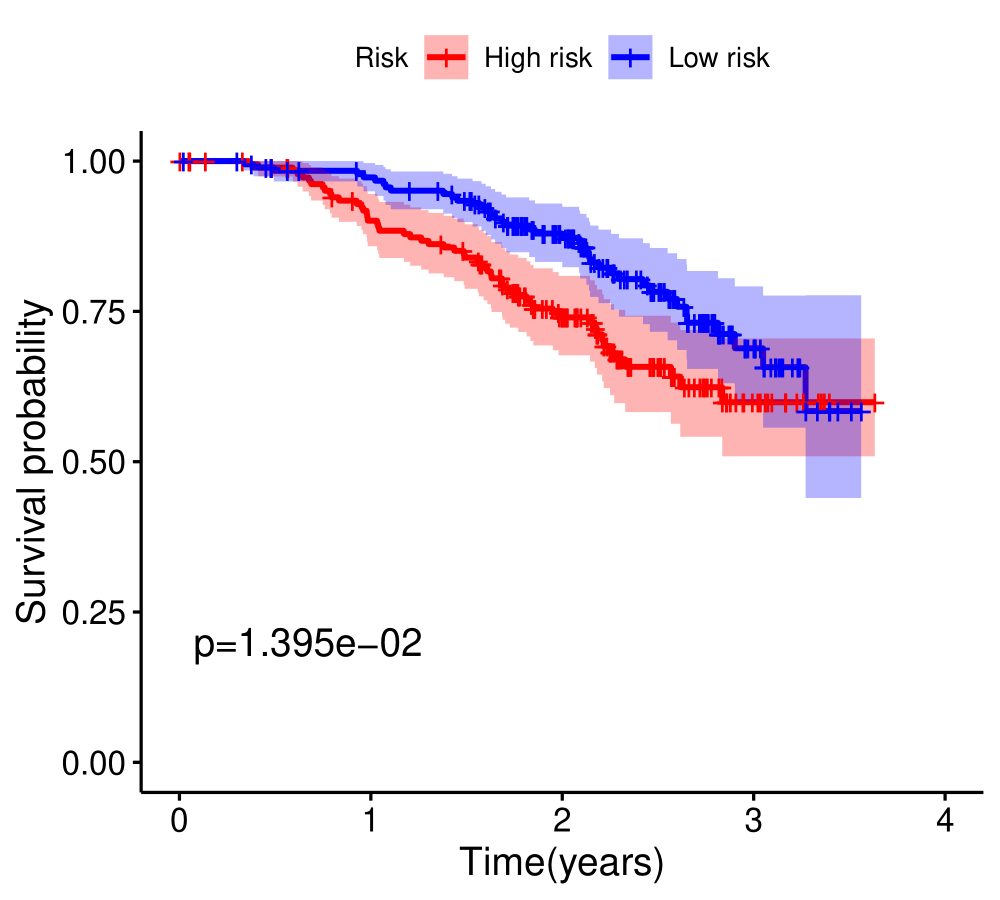

Supplement: Supplementary file 2 — Supplementary Figure 1. [file 41598_2024_61849_MOESM2_ESM.tiff]

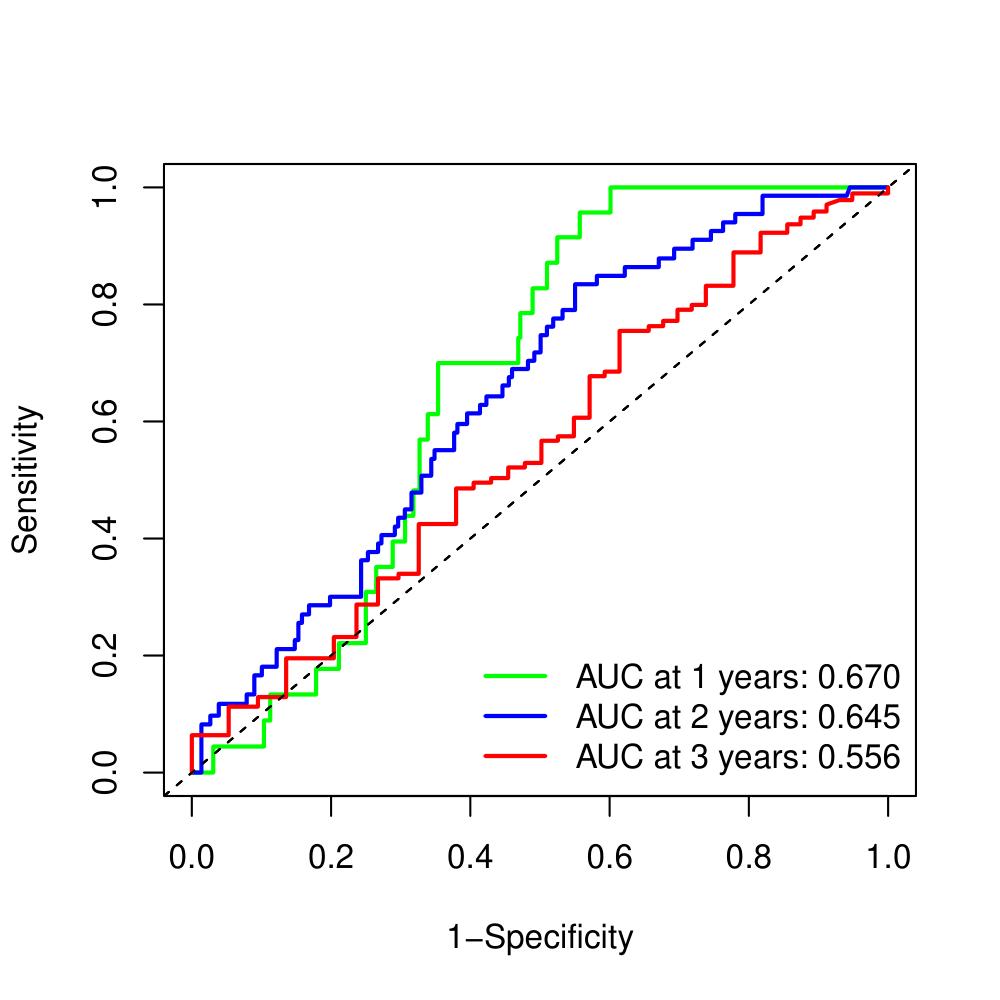

Supplement: Supplementary file 3 — Supplementary Figure 2. [file 41598_2024_61849_MOESM3_ESM.tiff]
